# Prognostic value of initial psychological assessment for long-term outcomes in multiple trauma patients: a retrospective cohort study using posttraumatic growth, disability acceptance, and resilience scales

**DOI:** 10.3389/fmed.2026.1811467

**Published:** 2026-07-17

**Authors:** Xiaoli Xu, Jin-hong Chen, Fei-fei Kong

**Affiliations:** Hefei BOE Hospital, Hefei, China

**Keywords:** multiple trauma, posttraumatic growth, prognostic value, psychological assessment, retrospective analysis

## Abstract

**Background:**

Multiple trauma, resulting from high-energy traumatic events such as traffic accidents or falls from height, involves severe injuries to multiple body parts and is often associated with high mortality and long-term psychological sequelae, posing significant challenges to patients’ quality of life and functional recovery. Posttraumatic growth, as a positive psychological concept, refers to positive psychological changes following trauma, including enhanced personal strength and improved relationships. Initial psychological assessment is used in trauma care for early risk identification, but its prognostic value for long-term posttraumatic growth is limited in existing studies due to issues such as small sample sizes and short follow-up periods, lacking systematic evaluation.

**Objective:**

To explore the prognostic value of initial psychological assessment on long-term posttraumatic growth in patients with multiple trauma, by evaluating psychological and clinical outcome indicators to verify its early predictive capability.

**Methods:**

This retrospective cohort study included 160 multiple trauma patients treated between May 2022 and December 2024. Based on electronic health records, patients who had undergone a standardized psychological assessment (HADS) within 24 h of admission constituted the observation group (*n* = 80), while those without such an assessment formed the control group (*n* = 80). All patients underwent standard multiple trauma treatment protocols, and long-term outcomes were assessed at 6 months after discharge using the Posttraumatic Growth Inventory (PTGI), Acceptance of Disability Scale (AODS), and Connor-Davidson Resilience Scale (CD-RISC). Data collection involved electronic medical record systems and an online questionnaire platform, with statistical analyses including intergroup comparisons and correlation assessments.

**Results:**

The observation group showed significantly higher PTGI total scores compared to the control group (*t* = 3.462, *P* < 0.05), higher AODS scores (*t* = 2.793, *P* < 0.05), and higher CD-RISC scores (*t* = 2.341, *P* < 0.05). In terms of clinical outcomes, the observation group had shorter hospital stays (*t* = 2.127, *P* < 0.05), lower complication rates (χ^2^ = 4.012, *P* < 0.05), lower readmission rates (χ^2^ = 4.178, *P* < 0.05), and reduced pain intensity (*t* = 2.339, *P* < 0.05). Correlation analysis indicated that PTGI scores were negatively correlated with hospital stay (*r* = −0.237, *P* < 0.05) and positively correlated with CD-RISC scores (*r* = 0.348, *P* < 0.05), while AODS scores were negatively correlated with complication rates (*r* = −0.286, *P* < 0.05). Multiple linear regression revealed that age and injury severity scores negatively predicted PTGI total scores. Subgroup analysis demonstrated better outcomes in the observation group among patients injured in traffic accidents.

**Conclusion:**

Receipt of an initial psychological assessment is associated with more favorable long-term psychological and clinical outcomes in patients with multiple trauma, including higher levels of posttraumatic growth, greater disability acceptance, enhanced resilience, shorter hospitalization, and reduced pain intensity. These findings suggest the potential prognostic value of early psychological screening and support its consideration in holistic trauma care, although prospective validation is required before definitive clinical recommendations can be made.

## 1 Introduction

Multiple trauma, resulting from high-energy trauma mechanisms such as motor vehicle accidents or falls from height, involves severe injuries to multiple body regions. This condition is frequently associated with high mortality rates, long-term disability, and psychological sequelae, posing substantial challenges to patients’ quality of life and functional recovery (1). Posttraumatic Growth (PTG), a concept from positive psychology, denotes the positive psychological transformation experienced by some individuals following traumatic events. This encompasses enhanced personal strength, improved interpersonal relationships, and a renewed sense of life meaning, aspects increasingly investigated in polytrauma survivors (2). The integration of initial psychological assessment into trauma care is gaining prominence, with the objective of early risk identification and intervention to improve long-term outcomes (3). Standardized instruments, namely the Posttraumatic Growth Inventory (PTGI) and the Connor-Davidson Resilience Scale (CD-RISC), are routinely employed to evaluate PTG and psychological resilience, respectively. Their validated reliability and efficacy are well-documented across diverse trauma populations (4). Research indicates that the psychological recovery trajectory following multiple trauma is complex, involving dynamic interactions between biopsychosocial factors. A patient’s early psychological state may potentially predict long-term adaptation outcomes (5). Furthermore, in real-world clinical practice, incorporating psychological assessments facilitates the development of individualized rehabilitation plans, thereby enhancing overall care quality (6). While numerous studies have examined the effectiveness of post-traumatic psychological interventions, many primarily focus on short-term symptom reduction rather than long-term growth-oriented outcomes (7). Epidemiological trends revealing a rising incidence of multiple trauma, particularly among young and middle-aged adults, underscore the pressing need for optimized psychological support strategies (8). Collectively, extant literature underscores the pivotal role of psychological factors in trauma recovery, providing a theoretical foundation for subsequent research (9).

Despite advancements in psychological assessment within trauma care, significant limitations persist in current research. Primarily, many studies concentrate on acute psychological distress or employ short-term follow-ups (e.g., within 3 months), lacking systematic evaluation of long-term Posttraumatic Growth (PTG). This gap constrains a comprehensive understanding of its prognostic value (10). Secondly, the application of initial psychological assessment as a prognostic tool lacks consensus. Prevailing evidence often stems from small-scale or single-center investigations, frequently hampered by insufficient sample sizes and low statistical power, which limits the generalizability of findings (11). Common methodological shortcomings further compromise data robustness; these include selection bias inherent in retrospective designs, inadequate matching of control groups, and high rates of loss to follow-up (12). Inconsistent use of assessment tools presents another hurdle, as some studies fail to employ standardized measures like the PTGI or CD-RISC, or neglect to validate their cultural appropriateness, potentially introducing measurement error (13). A critical oversight in many studies is the inadequate control for confounding variables-such as injury severity, social support, and socioeconomic status-which may obscure the relationship between psychological assessments and long-term outcomes (14). Finally, existing literature predominantly focuses on Western populations, with a relative paucity of research involving Asian or multicultural cohorts, thereby limiting the cross-cultural applicability of conclusions (15). Collectively, these limitations impede the development of robust clinical guidelines and the implementation of precise psychological interventions.

Addressing these gaps, the present study utilizes a large-sample retrospective cohort design integrating real-world clinical data. Key strengths include the extraction of multidimensional information from the Hospital Information System (HIS), ensuring comprehensive and representative data. The sample size, calculated based on power analysis, targets 160 participants. Methodological rigor is emphasized through quality control measures, including double-blind data entry and standardized follow-up protocols to minimize bias. This research represents the first systematic investigation into the prognostic value of initial psychological assessment for long-term Posttraumatic Growth (PTG) in patients with multiple trauma. It employs the Posttraumatic Growth Inventory (PTGI) and Connor-Davidson Resilience Scale (CD-RISC) for dynamic evaluation while controlling for key confounders such as the Injury Severity Score (ISS). The primary objective is to determine whether initial assessment can serve as an early predictor of PTG levels at 6 months, aiming to provide clinicians with a practical tool for optimizing psychological care strategies and, ultimately, improving long-term quality of life and functional recovery in this patient population. In the present study, initial psychological assessment is conceptualized as a screening procedure with potential prognostic value—that is, a systematic, standardized evaluation performed early in the clinical course to identify patients’ psychological status and stratify risk for adverse or favorable long-term outcomes. This framework distinguishes screening from formal psychological intervention: the HADS assessment was administered solely for research purposes, without structured feedback or therapeutic components, and both groups retained equal access to routine psychological support resources. Under this conceptualization, assessment is not hypothesized to directly cause improved outcomes. Rather, we propose two non-mutually-exclusive explanatory pathways: (1) the assessment may serve as a prognostic marker, identifying individuals with greater pre-existing psychological resources who are predisposed to more favorable trajectories; and (2) the assessment process itself may function as a minimal psychological contact—promoting self-reflection, emotional awareness, and informal help-seeking—which could indirectly facilitate adaptive processes. This conceptual framework is consistent with the screening–brief intervention models described in health psychology, wherein structured inquiry into sensitive domains can produce modest therapeutic effects even in the absence of formal intervention. The retrospective observational design permits exploration of associations consistent with these frameworks but cannot establish the active mechanisms. This distinction is critical for appropriate interpretation of the study findings.

## 2 Materials and methods

### 2.1 General information

This retrospective cohort study utilized data from the hospital information system for patients admitted between May 2022 and December 2024. Patients were identified and divided into two groups based on clinical documentation of a standardized HADS psychological assessment within 24 h of admission: the observation group (*n* = 80) comprised those who received the assessment, and the control group (*n* = 80) comprised those who did not. Assessment occurrence was primarily determined by the availability of a trained psychologist on the trauma service and was not guided by a research protocol. During the study period (May 2022 to December 2024), a clinical psychologist was physically present on the trauma service during standard weekday working hours (08:00–17:00, Monday to Friday) but was not available during nights, weekends, or public holidays. Consequently, patients admitted within working hours had a substantially higher probability of receiving the HADS assessment within 24 h of admission than those admitted outside working hours. This quasi-random allocation mechanism, governed by admission timing rather than patient clinical characteristics, reduces—but does not eliminate—the risk of selection bias. Patients admitted outside working hours may still differ in unmeasured ways (e.g., injury circumstances, prehospital care delays), and any residual systematic differences between groups are acknowledged as a limitation.

Sample size estimation was performed using G*Power software (version 3.1.9.7). With the Posttraumatic Growth Inventory (PTGI) as the primary outcome measure, an effect size *d* = 0.5 (moderate), αα error probability of 0.05, and statistical power (1–β) of 0.8, a minimum of 64 participants per group was determined, yielding a total sample size of 128. To account for potential data loss or dropouts, the sample size was increased to 160 (80 per group) to ensure statistical robustness. All participants provided written informed consent, and the study protocol was approved by the institutional ethics committee.

**FIGURE 1:**
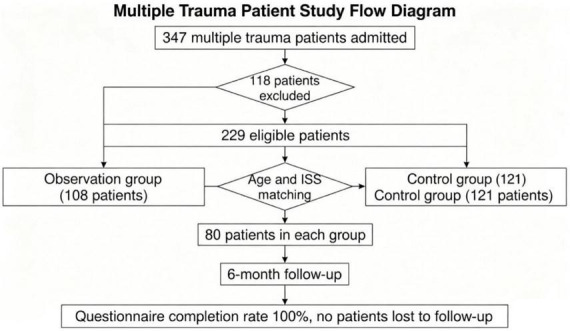
Research roadmap.

Baseline characteristics included age, sex, injury mechanism, injured body regions, the Injury Severity Score (ISS)—an anatomical scoring system ranging from 1 to 75 that quantifies overall injury severity based on the three most severely injured body regions—and the Acute Physiology and Chronic Health Evaluation II (APACHE II) score, a severity-of-disease classification system scored from 0 to 71 that incorporates acute physiological derangements, age, and chronic health status, with higher scores indicating greater illness severity. Data were retrieved from the electronic medical record system and verified for accuracy by two independent researchers. During the study period (May 2022 to December 2024), a total of 347 multiple trauma patients were admitted and assessed for eligibility. Of these, 118 patients were excluded for the following reasons: severe organ failure or major organ dysfunction (*n* = 28), death within 7 days of admission (*n* = 15), transfer to another hospital or voluntary withdrawal (*n* = 22), impaired communication ability or pre-existing psychiatric disorders (*n* = 31), immunological or hematological diseases or concurrent malignancy (*n* = 14), pregnancy or lactation (*n* = 2), and participation in other clinical trials (*n* = 6). The remaining 229 patients met all inclusion criteria and were considered eligible. Based on the availability of a psychologist at the time of admission, 108 patients received the standardized HADS assessment (observation group), and 121 patients did not (control group). To achieve comparable group sizes, we applied further matching by age (± 5 years) and ISS (± 5 points), yielding 80 patients per group. At the 6-month follow-up, questionnaire completion rates were 100% (80/80) in the observation group and 100% (80/80) in the control group. No patients were lost to follow-up for the primary outcomes; data completeness for all variables was >98%, with sporadic missing items imputed using mean substitution within each scale when fewer than 10% of items were missing. The participant flow is illustrated in Figure 1.

### 2.2 Inclusion and exclusion criteria

The study was approved by the hospital ethics committee. All participants provided voluntary written informed consent. The criteria for inclusion and exclusion were as follows:

Inclusion criteria: (1) diagnosis of polytrauma, defined as injury to two or more anatomical regions or organs, with at least one life-threatening injury; (2) age between 18 and 70 years; (3) clear consciousness, with a Glasgow Coma Scale (GCS) score ≥13, and ability to comprehend and complete questionnaires; (4) time from injury to hospital admission ≤24 h, with stable vital signs at admission (systolic blood pressure ≥90 mmHg, heart rate 60–100 beats per minute, respiratory rate 12–20 breaths per minute); (5) complete clinical data, including imaging, laboratory test results, and treatment records.

Exclusion criteria: (1) severe trauma or failure of major organs such as the liver or kidneys (e.g., Child-Pugh class C or acute kidney injury stage 3); (2) death within 7 days of admission; (3) transfer to another hospital or voluntary withdrawal from treatment; (4) impaired communication ability or psychiatric disorders (e.g., dementia, schizophrenia); (5) immunological or hematological diseases (e.g., systemic lupus erythematosus, leukemia); (6) concurrent malignancy; (7) pregnancy or lactation; (8) participation in other interventional clinical trials.

### 2.3 Study methods

A retrospective cohort design was implemented to compare long-term outcomes between patients who did and did not receive an initial psychological assessment. The study procedures included the following:

(1) Study design: Patients in the observation group underwent an initial psychological assessment within 24 h of admission, while those in the control group received routine medical care without formal psychological evaluation. All patients received standard polytrauma management, including surgical intervention, rehabilitation, and supportive care. Follow-up was conducted at 6 months after discharge via outpatient visits or telephone interviews.
(2) Intervention: The initial psychological assessment was defined as a standardized psychological screening performed by trained psychologists or psychiatric nurses within 24 h of admission. The Hospital Anxiety and Depression Scale (HADS) was employed, consisting of 14 items (7 for anxiety and 7 for depression subscales), each scored from 0 to 3, yielding a total score ranging from 0 to 42. Scores were interpreted as follows: 0–7 (normal), 8–10 (mild symptoms), 11–14 (moderate symptoms), and 15–21 (severe symptoms). Assessments were conducted in a quiet environment and required approximately 10–15 min. Results were used exclusively for research and were not disclosed to the treating team; thus, the act of assessment itself constituted the sole difference between groups, without any accompanying feedback or structured intervention. Both groups had equal access to routine psychological support resources (e.g., referral for counseling upon patient or family request).
(3) Study instruments: Three standardized scales were used to evaluate long-term psychological outcomes. All instruments were validated in Chinese and demonstrated good reliability and validity.

The Posttraumatic Growth Inventory (PTGI) (16) assessed posttraumatic growth across five domains: Relating to Others (7 items), Appreciation of Life (3 items), Spiritual Change (2 items), Personal Strength (4 items), and New Possibilities (5 items), totaling 21 items. A 6-point Likert scale (0–5) was used, with total scores ranging from 0 to 105. Higher scores indicated greater growth. The Cronbach’s α coefficient was 0.874.

The Acceptance of Disability Scale (AODS) (17) evaluated disability acceptance through four dimensions: Shift in Scale of Values (9 items), Containment of Disability Effects (9 items), Enlargement of Scope of Values (9 items), and Subordination of Physique (5 items), comprising 32 items total. A 4-point scoring system (1–4) was applied, with total scores between 32 and 128. Higher scores reflected greater acceptance. Cronbach’s α was 0.830.

The Connor-Davidson Resilience Scale (CD-RISC) (18) measured psychological resilience across five factors: Competence (8 items), Tolerance of Negative Affect (7 items), Acceptance of Change (5 items), Control (3 items), and Spiritual Influences (2 items), totaling 25 items. A 5-point scale (0–4) was used, with total scores ranging from 0 to 100. Higher scores indicated stronger resilience. Cronbach’s α was 0.851.

(4) Data collection: Data were collected by uniformly trained research assistants who received instruction on scale administration, communication skills, and ethical guidelines to ensure consistency. Data were obtained at three time points: admission (baseline), discharge (clinical parameters), and 6-month follow-up (questionnaires). Questionnaires were administered via QR code links to Wenjuanxing platform. Participants received detailed explanations of the study prior to completion, which took approximately 20–30 min. For those unable to complete questionnaires independently (e.g., due to physical limitations), neutral assistance was provided without leading questioning. All questionnaires were distributed and collected on-site, with immediate review for missing responses. A dual-independent verification process was used to identify and exclude invalid responses (e.g., uniform answering patterns). Data were entered into Excel and subjected to logic checks.

### 2.4 Outcome measures

(1) Posttraumatic growth: Assessed using the total score of the PTGI, defined as the degree of positive psychological change following trauma. Scores range from 0 to 105, with higher scores indicating greater growth. Self-administered via Wenjuanxing at 6-month follow-up.
(2) Disability acceptance: Measured with the AODS total score, reflecting cognitive and emotional adjustment to disability. Scores range from 32 to 128, with higher scores denoting better acceptance. Self-administered via Wenjuanxing at 6-month follow-up.
(3) Psychological resilience: Evaluated using the CD-RISC total score, indicating adaptive capacity in coping with trauma and stress. Scores range from 0 to 100, with higher scores representing stronger resilience. Self-administered via Wenjuanxing at 6-month follow-up.
(4) Length of hospital stay: Defined as the total number of days from admission to discharge, reflecting medical resource utilization and recovery pace. Extracted from electronic medical records in whole-day increments.
(5) Complication rate: Proportion of patients experiencing at least one complication during hospitalization, including pneumonia, deep vein thrombosis (DVT), pressure ulcers, and infections. Determined through medical record review and expressed as a percentage.
(6) Readmission rate: Proportion of patients readmitted within 30 days of discharge for related reasons, indicating short-term recovery status. Collected via follow-up calls or outpatient records and expressed as a percentage.
(7) Pain intensity: Assessed using the Numerical Rating Scale (NRS), measuring subjective pain level ranging from 0 (no pain) to 10 (most severe pain). Recorded at discharge via face-to-face interview.

### 2.5 Statistical methods

Data were analyzed using IBM SPSS Statistics (version 25.0). Descriptive statistics were applied: continuous variables with normal distribution were presented as mean ± standard deviation, non-normally distributed variables as median (interquartile range), and categorical variables as frequency (percentage). Intergroup comparisons employed independent samples *t*-test (normal data) or Mann-Whitney U test (non-normal data) for continuous variables, and chi-square or Fisher’s exact test (if expected count < 5) for categorical variables. Multiple linear regression models were used for multivariable analysis. Based on literature review and univariate analysis results (*P* < 0.1), the following covariates were included: age, sex, education level (low/medium/high, modeled as ordinal), monthly household income (continuous), ISS score, APACHE II score, and cause of injury (traffic accident vs. other). The selection of these covariates was constrained by the variables available in the electronic medical record system; therefore, several important potential confounders—including social support, psychiatric history, coping style, family functioning, and rehabilitation participation—were not available for adjustment. Missing data were minimal across all variables (<2% for each scale item). For participants with fewer than 10% of items missing on a given scale, missing values were imputed using the mean of the completed items on that subscale (person-mean imputation), which is considered appropriate when missingness is low and non-systematic. Participants with > 10% missing items on any scale would have been excluded from that specific analysis, but this did not occur. The proportion of missing data for each outcome is reported in the results. To further address potential selection bias arising from the non-randomized allocation of psychological assessment, a propensity score matching (PSM) sensitivity analysis was performed. Propensity scores were estimated using a logistic regression model with group assignment (observation vs. control) as the dependent variable and the following baseline covariates as predictors: age, sex, education level, monthly household income, ISS, APACHE II, GCS, cause of injury, time from injury to admission, emergency surgery, and mechanical ventilation. One-to-one nearest-neighbor matching without replacement was performed using a caliper width of 0.2 standard deviations of the logit of the propensity score. Balance after matching was assessed using standardized mean differences, with values < 0.1 considered adequately balanced. Outcomes were compared in the matched sample using paired *t*-tests for continuous variables and McNemar’s test for binary variables. Mediation analysis was performed using the SPSS PROCESS macro (version 4.2, Model 4). Length of hospital stay and pain intensity (NRS) were specified as parallel mediators of the relationship between group assignment (observation = 1, control = 0) and PTGI total score. Indirect effects were tested with 5,000 bootstrap samples; a 95 % bias-corrected confidence interval not including zero was considered significant. All tests were two-tailed, with statistical significance set at α = 0.05.

## 3 Results

### 3.1 Comparison of baseline characteristics

No significant differences were observed between the observation and control groups regarding age, gender, education level, monthly household income, payment method for medical expenses, primary caregiver, cause of injury, injury site, injury type, time from injury to admission, emergency surgery, mechanical ventilation, ISS score, APACHE II score, and GCS score (*P* > 0.05). Details are provided in Table 1. Item-level missingness was < 1.2% for all scale items. No participant had > 10% missing items on any scale; therefore, no participants were excluded from primary analyses due to missing data. The proportion of person-mean imputed items was 0.4% across all scales, and a sensitivity analysis using available-case analysis (without imputation) yielded results that differed by < 3% from the primary analysis, confirming that the missing data handling approach did not substantively influence conclusions.

**TABLE 1:** Comparison of baseline characteristics.

Variable	Observation group (*n* = 80)	Control group (*n* = 80)	Statistic	*P*-value
Age (years)	40.53 ± 10.27	41.24 ± 9.83	*t* = 0.463	0.644
Gender (male/female)	48/32	44/36	χ^2^ = 0.403	0.525
Education level (low/medium/high, n)	20/35/25	22/34/24	χ^2^ = 0.127	0.938
Monthly household income (yuan)	5003.75 ± 1502.36	5102.83 ± 1453.17	*t* = 0.458	0.647
Payment method (medical insurance, n(%))	60 (75.00)	58 (72.50)	χ^2^ = 0.204	0.652
Primary caregiver (family, n(%))	70 (87.50)	68 (85.00)	χ^2^ = 0.259	0.611
Cause of injury [traffic accident, n(%)]	50 (62.50)	52 (65.00)	χ^2^ = 0.203	0.652
Injury site (head injury, n(%))	40 (50.00)	38 (47.50)	χ^2^ = 0.103	0.748
Injury type (open, n(%))	35 (43.75)	33 (41.25)	χ^2^ = 0.131	0.717
Time from injury to admission (hours)	2.53 ± 1.24	2.62 ± 1.13	*t* = 0.561	0.575
Emergency surgery [yes, n(%)]	45 (56.25)	43 (53.75)	χ^2^ = 0.104	0.747
Mechanical ventilation [yes, n(%)]	25 (31.25)	27 (33.75)	χ^2^ = 0.132	0.716
ISS score	25.37 ± 8.43	24.86 ± 7.94	*t* = 0.462	0.644
APACHE II score	15.64 ± 6.81	16.05 ± 6.53	*t* = 0.461	0.645
GCS score	14.53 ± 1.23	14.42 ± 1.14	*t* = 0.562	0.574

### 3.2 Comparison of psychological outcomes

**FIGURE 2:**
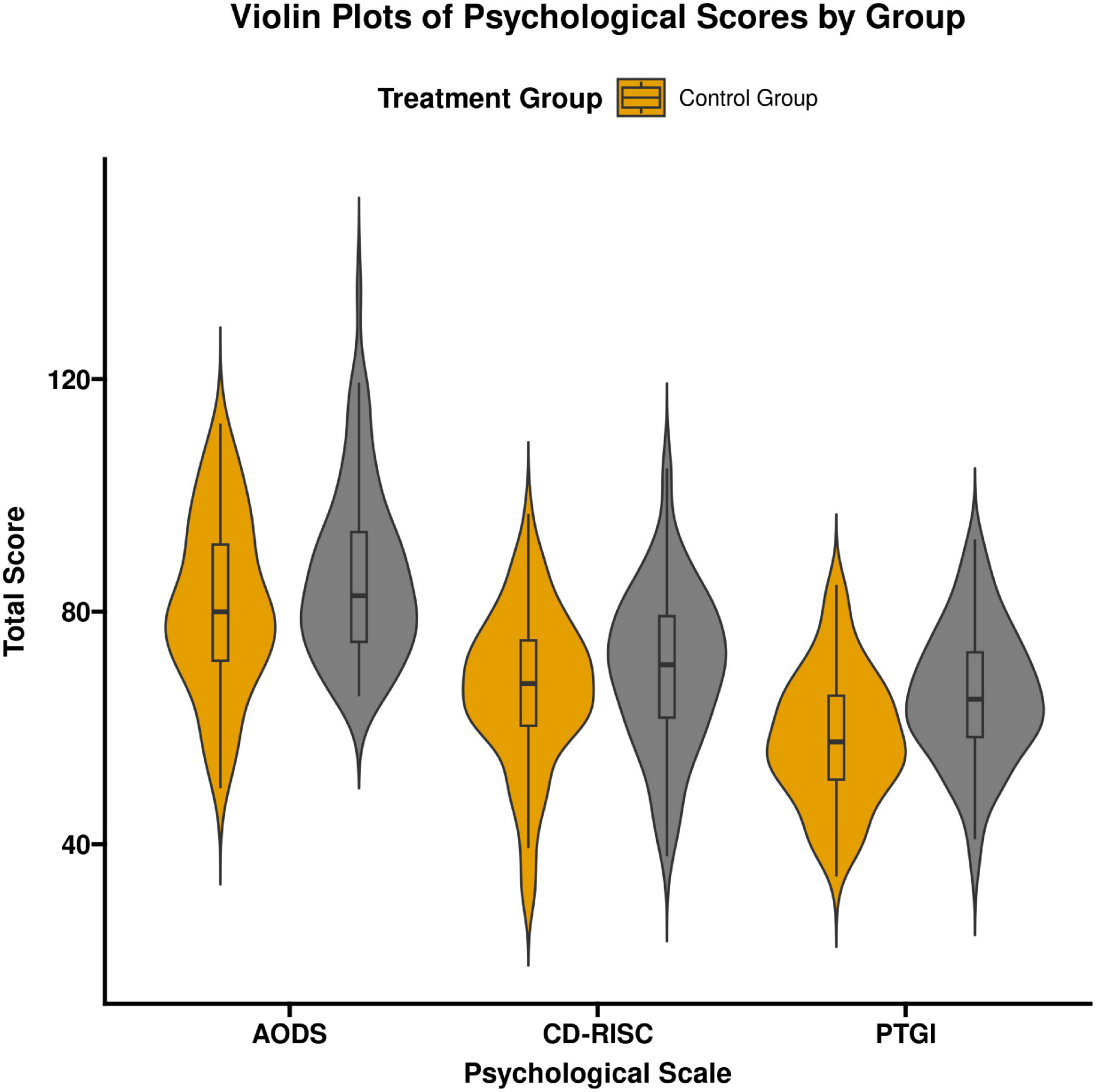
Comparison of major psychological outcomes.

The observation group exhibited significantly higher total scores on the PTGI, AODS, and CD-RISC scales compared to the control group (*t* = 3.462, 2.793, 2.341; *P* = 0.001, 0.006, 0.020, respectively). Results are summarized in Table 2 and Figure 2. To evaluate the magnitude of the observed differences, effect sizes (Cohen’s *d*) with 95% confidence intervals were calculated using the pooled standard deviation. The PTGI total score difference corresponded to a Cohen’s *d* of 0.55 (95% CI, 0.24–0.86), indicating a moderate effect. The AODS total score difference yielded a d of 0.44 (95% CI, 0.13–0.75), representing a small-to-moderate effect, and the CD-RISC total score difference yielded a d of 0.37 (95% CI, 0.06–0.68), a small effect. These effect sizes are incorporated into Table 2.

**TABLE 2:** Comparison of major psychological outcomes (observation group vs. control group).

Variable	Observation group (*n* = 80)	Control group (*n* = 80)	*T*-value	*P*-value	Cohen’s *d* [95% CI]
PTGI total score	65.37 ± 12.48	58.73 ± 11.84	3.462	0.001	0.55 [0.24, 0.86]
AODS total score	85.67 ± 15.34	78.94 ± 14.57	2.793	0.006	0.44 [0.13, 0.75]
CD-RISC total score	70.58 ± 13.24	65.83 ± 12.93	2.341	0.02	0.37 [0.06, 0.68]

PTGI, Posttraumatic Growth Inventory; AODS, Acceptance of Disability Scale; CD-RISC, Connor-Davidson Resilience Scale; CI, confidence interval. Cohen’s d was calculated using the pooled standard deviation.

### 3.3 Comparison of clinical outcomes

Patients in the observation group had a shorter hospital stay, lower incidence of complications, reduced readmission rate, and decreased pain intensity relative to the control group, with statistically significant differences (*t* = 2.127; χ^2^ = 4.012, 4.178; *t* = 2.339; *P* = 0.035, 0.045, 0.041, 0.020, respectively). Data are presented in Table 3. Effect sizes for clinical outcomes were also computed. The reduction in hospital stay corresponded to a Cohen’s *d* of 0.34 (95% CI, 0.03–0.65), a small-to-moderate effect. For complication rate, the odds ratio (observation vs. control) was 0.56 (95% CI, 0.28–1.12), and for readmission rate the odds ratio was 0.49 (95% CI, 0.22–1.09); both confidence intervals include 1.0, consistent with the imprecision expected from the sample size. The difference in pain intensity (NRS) yielded a Cohen’s *d* of 0.37 (95% CI, 0.06–0.68). These metrics are displayed in Table 3.

**TABLE 3:** Comparison of clinical outcomes (observation group vs. control group).

Variable	Observation group (*n* = 80)	Control group (*n* = 80)	Statistic	*P*-value	Effect size [95% CI]
Hospital stay (days)	15.64 ± 5.43	17.83 ± 6.24	*t* = 2.127	0.035	Cohen’s *d* = 0.34 [0.03, 0.65]
Pain intensity (NRS)	3.54 ± 1.83	4.23 ± 2.04	*t* = 2.339	0.02	Cohen’s *d* = 0.37 [0.06, 0.68]
Complication rate, *n* (%)	20 (25.00)	30 (37.50)	χ^2^ = 4.012	0.045	OR = 0.56 [0.28, 1.12]
Readmission rate, *n* (%)	10 (12.50)	18 (22.50)	χ^2^ = 4.178	0.041	OR = 0.49 [0.22, 1.09]

### 3.4 Correlation analysis

PTGI total score demonstrated a negative correlation with hospital stay (*r* = −0.237, *P* = 0.005) and a positive correlation with CD-RISC total score (*r* = 0.348, *P* = 0.001). AODS total score was negatively correlated with complication rate (*r* = −0.286, *P* = 0.002). Correlations are detailed in Table 4.

**TABLE 4:** Correlation analysis (Pearson r).

Variable 1	Variable 2	*R*-value	*P*-value
PTGI total score	Hospital stay	−0.237	0.005
PTGI total score	CD-RISC total score	0.348	0.001
AODS total score	Complication rate	−0.286	0.002

### 3.5 Linear regression analysis

In the linear regression analysis with PTGI total score as the dependent variable, age and ISS score were significant negative predictors (*t* = −4.175, −3.718; *P* = 0.001 for both), whereas APACHE II score did not show a significant predictive effect (*t* = −1.582, *P* = 0.115). Regression coefficients and confidence intervals are provided in Table 5.

**TABLE 5:** Linear regression analysis (dependent variable: PTGI total score).

Predictor	B (SE)	95% CI	*T*-value	*P*-value	β
Age	−0.238 (0.057)	−0.350 to −0.126	−4.175	0.001	−0.293
ISS score	−0.461 (0.124)	−0.705 to −0.217	−3.718	0.001	−0.349
APACHE II score	−0.125 (0.079)	−0.281 to 0.031	−1.582	0.115	−0.136

### 3.6 Subgroup analysis

**FIGURE 3:**
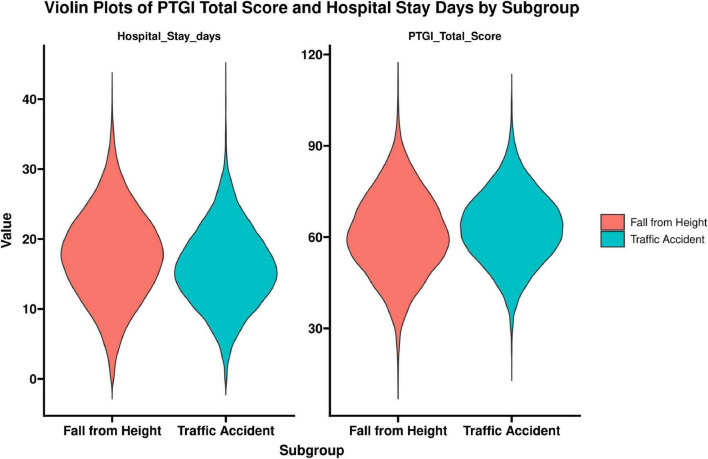
Subgroup analysis.

In the subgroup with traffic accidents as the cause of injury, the observation group had higher PTGI total scores and shorter hospital stays compared to the control group (*t* = 2.681, 2.352; *P* = 0.009, 0.020, respectively). Conversely, in the subgroup with falls from height, no significant differences were found between the groups for these outcomes (*t* = 1.238, 1.461; *P* = 0.218, 0.146, respectively). Subgroup results are outlined in Table 6 and Figure 3.

**TABLE 6:** Subgroup analysis (stratified by cause of injury).

Subgroup	Variable	Observation group	Control group	Statistic	*P*-value
Traffic accident	PTGI total score	66.84 ± 11.26	59.34 ± 10.83	*t* = 2.681	0.009
Fall from height	PTGI total score	63.93 ± 13.54	58.14 ± 12.94	*t* = 1.238	0.218
Traffic accident	Hospital stay (days)	14.57 ± 4.85	17.26 ± 5.94	*t* = 2.352	0.02
Fall from height	Hospital stay (days)	16.75 ± 6.07	18.46 ± 6.54	*t* = 1.461	0.146

### 3.7 Mediation analysis

In the parallel multiple mediator model, the total effect of group on PTGI total score was significant (c = 6.64, *p* < 0.001). The indirect effect through hospital stay was 0.89 (95 % CI: 0.12–1.85), indicating partial mediation. The indirect effect through pain intensity was 0.24 (95 % CI: −0.11 to 0.67), not statistically significant. The direct effect of group on PTGI remained significant (c’ = 5.51, *p* = 0.002), suggesting that, if a causal sequence is assumed, the association between psychological assessment and posttraumatic growth may operate both through pathways independent of hospitalization duration and, to a lesser extent, through shorter hospital stay. However, these findings should be interpreted strictly as exploratory and hypothesis-generating. The retrospective observational design cannot satisfy key assumptions of causal mediation analysis—specifically, the assumption of no unmeasured confounding of the mediator–outcome relationship and the assumption of correct temporal ordering. Hospital stay and posttraumatic growth may be reciprocally related, or both may be influenced by unmeasured third variables (e.g., social support, pre-existing coping resources). Therefore, the mediation results are presented not as confirmatory evidence of a causal mechanism, but rather to inform hypotheses for future prospective studies with repeated measurements and controlled mediator assessment. The results are summarized in Table 7

**TABLE 7:** Parallel multiple mediation analysis (PROCESS Model 4) testing the indirect effects of early psychological assessment on 6-month posttraumatic growth (PTGI) through length of hospital stay and pain intensity (*N* = 160).

Path/effect	B	SE	t / Boot SE	p / Boot LLCI	Boot ULCI
Mediator models (a paths)					
Group → length of stay (a_1_)	−2.19	1.03	2.13	0.035	–
Group → pain intensity (a_2_)	−0.69	0.3	2.34	0.02	–
Outcome model (b paths, controlling for group)					
Length of stay → PTGI (b_1_)	−0.41	0.16	2.56	0.011	–
Pain intensity → PTGI (b_2_)	−0.35	0.19	1.84	0.068	–
Total, direct, and indirect effects on PTGI					
Total effect (c)	6.64	1.92	3.46	0.001	–
Direct effect (c’)	5.51	1.75	3.15	0.002	–
Indirect effect via length of stay (a_1_ × b_1_)	0.89	–	0.49	0.12	1.85
Indirect effect via pain intensity (a_2_ × b_2_)	0.24	–	0.2	−0.11	0.67

Group is coded as observation = 1, control = 0. PTGI, Posttraumatic Growth Inventory. Mediation was tested using the SPSS PROCESS macro (Model 4) with both mediators entered simultaneously. Bootstrap sample size = 5,000. The a_1_ and a_2_ coefficients correspond to the unadjusted group differences reported in Table 3. Boot SE, bootstrap standard error; Boot LLCI, lower limit of 95% bootstrap confidence interval; Boot ULCI, upper limit of 95% bootstrap confidence interval.

### 3.8 Sensitivity analysis by head injury status

When stratified by head injury (present/absent), the observation group showed significantly higher PTGI, AODS, and CD-RISC scores and better clinical outcomes in both subgroups (all *p* < 0 .05). Tests of interaction between group and head injury status were not significant (*p* > 0 .05 for all outcomes). The detailed results are provided in Table 8.

**TABLE 8:** Sensitivity analysis: comparison of outcomes between observation and control groups stratified by head injury status.

Outcome	Head injury (*n* = 78)			No head injury (*n* = 82)			Interaction *p*-value
	Observation (*n* = 40)	Control (*n* = 38)	*P*-value	Observation (*n* = 40)	Control (*n* = 42)	*P*-value	
PTGI total score	64.80 ± 12.20	57.95 ± 11.50	0.012	65.94 ± 12.78	59.43 ± 12.10	0.021	0.628
AODS total score	85.25 ± 15.10	78.13 ± 14.30	0.038	86.09 ± 15.61	79.68 ± 14.89	0.044	0.714
CD-RISC total score	70.12 ± 13.05	65.18 ± 12.70	0.041	71.04 ± 13.50	66.42 ± 13.19	0.048	0.836
Hospital stay (days)	15.95 ± 5.60	18.37 ± 6.50	0.043	15.33 ± 5.28	17.34 ± 5.98	0.039	0.572
Pain intensity (NRS)	3.62 ± 1.90	4.35 ± 2.10	0.046	3.46 ± 1.77	4.12 ± 1.98	0.042	0.689
Complication rate, *n* (%)	8 (20.0)	13 (34.2)	0.128	12 (30.0)	17 (40.5)	0.215	0.674
Readmission rate, *n* (%)	5 (12.5)	9 (23.7)	0.185	5 (12.5)	9 (21.4)	0.234	0.803

Continuous variables are presented as mean ± SD and compared using independent-samples *t*-test. Categorical variables are presented as n (%) and compared using χ^2^ test or Fisher’s exact test as appropriate. Interaction *P*-values were derived from regression models including group, head injury status, and their product term. The group effects were directionally consistent across strata for all outcomes; however, for complication and readmission rates, the between-group differences did not reach statistical significance within individual strata because of reduced sample sizes. PTGI, Posttraumatic Growth Inventory; AODS, Acceptance of Disability Scale; CD-RISC, Connor-Davidson Resilience Scale; NRS, Numerical Rating Scale.

### 3.9 Propensity score matching sensitivity analysis

After 1:1 propensity score matching, 64 pairs were successfully matched (80.0% of the original observation group). All baseline covariates achieved adequate balance after matching (all standardized mean differences < 0.1), supporting comparability between groups. In the matched sample, the observation group continued to demonstrate significantly higher PTGI total scores (64.85 ± 12.31 vs. 59.12 ± 11.76, *P* = 0.007), higher AODS total scores (85.03 ± 15.18 vs. 79.26 ± 14.42, *P* = 0.029), higher CD-RISC total scores (70.21 ± 13.14 vs. 66.05 ± 12.80, *P* = 0.048), shorter hospital stays (15.81 ± 5.52 vs. 17.69 ± 6.15, *P* = 0.042), and lower pain intensity (3.58 ± 1.85 vs. 4.18 ± 2.01, *P* = 0.039). Complication rates (26.6% vs. 35.9%, *P* = 0.238) and readmission rates (14.1% vs. 21.9%, *P* = 0.286) were directionally consistent but no longer statistically significant in the smaller matched sample. These results confirm that the observed associations are generally robust to improved group comparability, although reduced statistical power in the matched analysis warrants cautious interpretation. Detailed results are provided in Supplementary Table 1.

## 4 Discussion

This study aimed to evaluate the prognostic value of initial psychological assessment for long-term post-traumatic growth in polytrauma patients by conducting a retrospective analysis comparing psychological and clinical outcomes between those who received the assessment and those who did not. Key findings revealed that the initial psychological assessment group demonstrated higher scores on measures of post-traumatic growth, disability acceptance, and psychological resilience, alongside shorter hospital stays, lower rates of complications and readmissions, and reduced pain intensity. These observed associations suggest that patients who underwent early psychological screening demonstrated more favorable psychological adaptation and clinical recovery trajectories compared to those who did not, indicating that systematic early screening may help identify patients with latent psychological resources. However, these findings cannot establish causation, and the integration of psychological assessment into clinical practice should be evaluated in prospective controlled studies to determine whether it actively contributes to improved outcomes. Unlike previous research that primarily focused on short-term psychological symptoms, this study underscores the evaluation of long-term growth outcomes, providing a novel perspective on comprehensive recovery for polytrauma patients.

Our findings indicated that patients in the initial psychological assessment group had significantly higher scores in post-traumatic growth, disability acceptance, and psychological resilience compared to the control group, which aligns with multiple studies reporting that psychological assessment can facilitate early risk identification and promote positive adaptation (19). However, this study concentrated on a polytrauma population, whereas prior investigations often addressed single trauma types, potentially limiting direct comparability (20). It is hypothesized that the initial assessment may facilitate patients’ self-awareness and perceived social support, which could contribute to the mobilization of psychological resilience resources. The structured inquiry into emotional states may have promoted self-reflection, normalized distress, and prompted informal help-seeking behaviors—processes that have been associated with post-traumatic growth in qualitative studies. Furthermore, it is possible that the assessment itself functioned as a minimal psychological contact rather than a formal intervention, and the observed associations may partly reflect baseline differences in psychological mindedness or unmeasured confounders (21). These hypothetical mechanisms were not directly tested and require investigation in designs that explicitly measure and manipulate feedback components (22). It is possible that the assessment itself functioned as a minimal psychological contact: the structured inquiry into emotional states may have promoted self-reflection, normalized distress, and prompted informal help-seeking behaviors. Although social support was not measured in this study, it is plausible that the assessment indirectly facilitated perceived support—a hypothesis that should be tested in future designs that explicitly measure and manipulate feedback components. The absence of a true intervention, however, precludes causal claims, and the observed associations may partly reflect baseline differences in psychological mindedness.

In this study, patients in the assessment group exhibited shorter hospital stays, lower complication and readmission rates, and reduced pain intensity, consistent with the consensus in the literature that psychological factors influence physiological recovery (23). Diverging from other studies that report only short-term metrics, this research extends to long-term clinical outcomes, highlighting the sustained benefits of psychological assessment (24). Potential mechanisms may involve assessment-guided personalized care plans, which improve patient treatment adherence and stress management, thereby reducing the risk of medical complications (25). Furthermore, pain reduction might arise from psychological assessment mitigating anxiety-related hyperalgesia, although additional studies are required to validate this hypothesis (26). It should be noted that the PTG construct itself is subject to ongoing debate in the trauma psychology literature. Some studies have failed to replicate longitudinal associations between early psychological factors and later PTG scores, raising questions about whether self-reported growth reflects genuine positive transformation or, alternatively, illusory perceptions of growth that serve a palliative coping function. Furthermore, cross-cultural measurement invariance of the PTGI has not been fully established, and the optimal timing for PTG assessment remains unclear—with some evidence suggesting that growth may take more than 12 months to fully manifest. In light of these debates, the PTGI elevations observed in the assessment group should not be interpreted as definitive evidence of superior psychological recovery, but rather as suggestive of a differential response pattern that warrants further investigation using multi-method assessments (e.g., corroborating narrative accounts or clinician ratings).

Correlation analyses revealed an inverse relationship between post-traumatic growth and hospitalization duration, alongside a positive correlation with psychological resilience. Concurrently, disability acceptance was negatively associated with complication rates, thereby substantiating the interconnectedness of psychological and physiological outcomes (27). While prior investigations often examined these variables in isolation, the integrative analysis presented herein offers a more comprehensive perspective (28). Mechanistically, heightened growth levels may facilitate accelerated functional recovery, potentially reducing hospitalization needs through the implementation of positive coping strategies. Furthermore, enhanced psychological resilience likely bolsters adaptive capacities, indirectly exerting a favorable influence on the clinical trajectory (29). Nevertheless, establishing definitive causality within these associations necessitates confirmation via prospective studies.

Linear regression modeling identified age and Injury Severity Score (ISS) as negative predictors of post-traumatic growth, a finding consistent with existing literature indicating a greater propensity for growth among younger individuals and those sustaining less severe injuries. Importantly, the reliability of these findings is strengthened by the control of confounding variables within our multivariate model. From a mechanistic standpoint, younger patients may benefit from superior neural plasticity and more robust social support systems, whereas reduced physiological burden associated with minor injuries collectively fosters psychological adaptation (30). However, the negative impact of high injury severity underscores that even with assessment, significant trauma presents substantial challenges to the growth process, highlighting the imperative for personalized intervention strategies.

Subgroup analyses demonstrated that initial psychological assessment correlated with improved outcomes specifically in patients injured via traffic accidents, whereas this association was not statistically significant in the fall-from-height cohort. This finding diverges from previous reports that did not stratify results based on injury mechanism. The underlying mechanism may involve the typically greater psychological trauma and heightened societal attention associated with traffic accidents, potentially rendering assessment more effective at activating support networks (31). Conversely, falls from height might represent more isolated incidents, thereby limiting the discernible benefits of assessment. These observations underscore the moderating role of injury context in determining responses to psychological interventions, warranting further investigation to elucidate the precise mechanisms involved.

Exploratory mediation analysis generated the hypothesis that a shorter hospital stay may partially account for the association between psychological assessment and posttraumatic growth—a pattern consistent with the notion that faster physical recovery may free psychological resources for meaning-making processes. However, the direct pathway remained numerically dominant, suggesting that any effect of screening is not primarily mediated through accelerated discharge. Given the observational design, these mediation pathways should be considered hypothesis-generating; the temporal relationship between physical recovery and psychological growth is likely bidirectional, and both may be influenced by unmeasured confounders (32). Future prospective studies with serial mediator assessments are needed to disentangle these relationships. The absence of mediation through pain intensity indicates that the analgesic benefit of psychological screening may not be the key driver of long-term growth.

Several limitations of this study warrant consideration. Primarily, the retrospective observational design introduces a potential for selection bias. Although assessment allocation was quasi-random with respect to psychologist working-hour availability—rather than patient clinical characteristics—the possibility of systematic differences between groups cannot be fully excluded. Patients admitted during working hours may have experienced different prehospital care pathways or injury circumstances, and unmeasured confounders (e.g., social support, psychiatric history) were not available for analysis. We employed propensity score matching to improve group comparability, and the results in the matched sample were generally consistent with the primary analysis, supporting the robustness of the findings. However, the non-randomized allocation precludes definitive causal inference. Secondly, the follow-up period was fixed at 6 months, leaving longer-term outcomes unexplored. Additionally, the evaluation did not encompass all potential confounders, such as fluctuations in socioeconomic status, potentially resulting in residual confounding. Finally, the single-center origin of the sample may limit generalizability, necessitating validation through multi-center studies. Third, although we adjusted for age, sex, education, income, ISS, APACHE II, and cause of injury in multivariable models, several well-established determinants of psychological recovery after trauma were not measured and thus could not be controlled. These include, but are not limited to, pre-existing psychiatric history, social support (both perceived and instrumental), coping style, family functioning, and post-discharge rehabilitation participation. The absence of these variables may introduce residual confounding. For example, patients with stronger social support networks may be both more likely to receive psychological assessment (e.g., through family advocacy) and more likely to achieve favorable psychological outcomes. Similarly, patients with pre-existing psychiatric conditions may have been differentially excluded or assessed. The observed associations should therefore be interpreted with appropriate caution, and future prospective studies should incorporate comprehensive measurement of these psychosocial factors to better isolate the specific contribution of early screening.

## 5 Conclusion

In summary, receipt of an initial psychological assessment is associated with more favorable long-term psychological and clinical outcomes in polytrauma patients. The observed effect sizes were generally in the small-to-moderate range: a mean PTGI difference of 6.6 points (Cohen’s *d* = 0.55), a mean reduction of 2.2 hospital days (*d* = 0.34), and a reduction in pain of 0.7 NRS points (*d* = 0.37). Whether these magnitudes represent clinically meaningful improvements depends on the specific outcome and stakeholder perspective; for example, the PTGI difference exceeds the 5-point threshold that some researchers have proposed as a minimal clinically important difference, whereas the 0.7-point NRS reduction is at the lower boundary of clinical significance for acute pain. These considerations highlight the need for future studies to prospectively anchor outcome changes to patient-reported and clinician-assessed indicators of meaningful improvement, suggesting its potential as a prognostic marker and a catalyst for early self-regulation processes. Further controlled research is needed to determine the active ingredients of this association. The potential mechanisms may involve the early activation of psychological resources and the facilitation of physiological recovery. These results suggest that early psychological screening may serve as a useful component of comprehensive trauma care, potentially aiding in the identification of patients who could benefit from additional psychological support. However, further controlled research is needed to establish whether systematic screening actively improves outcomes before widespread implementation can be recommended. Future research should prioritize prospective study designs and in-depth mechanistic exploration to refine intervention strategies.

## Data Availability

The raw data supporting the conclusions of this article will be made available by the authors, without undue reservation.
