# Supplementary figures and images for: Prognostic value of initial psychological assessment for long-term outcomes in multiple trauma patients: a retrospective cohort study using posttraumatic growth, disability acceptance, and resilience scales
Source: Front Med (Lausanne). 2026 Jul 17;13:1811467. doi: 10.3389/fmed.2026.1811467 (PMC13423887; doi:10.3389/fmed.2026.1811467)

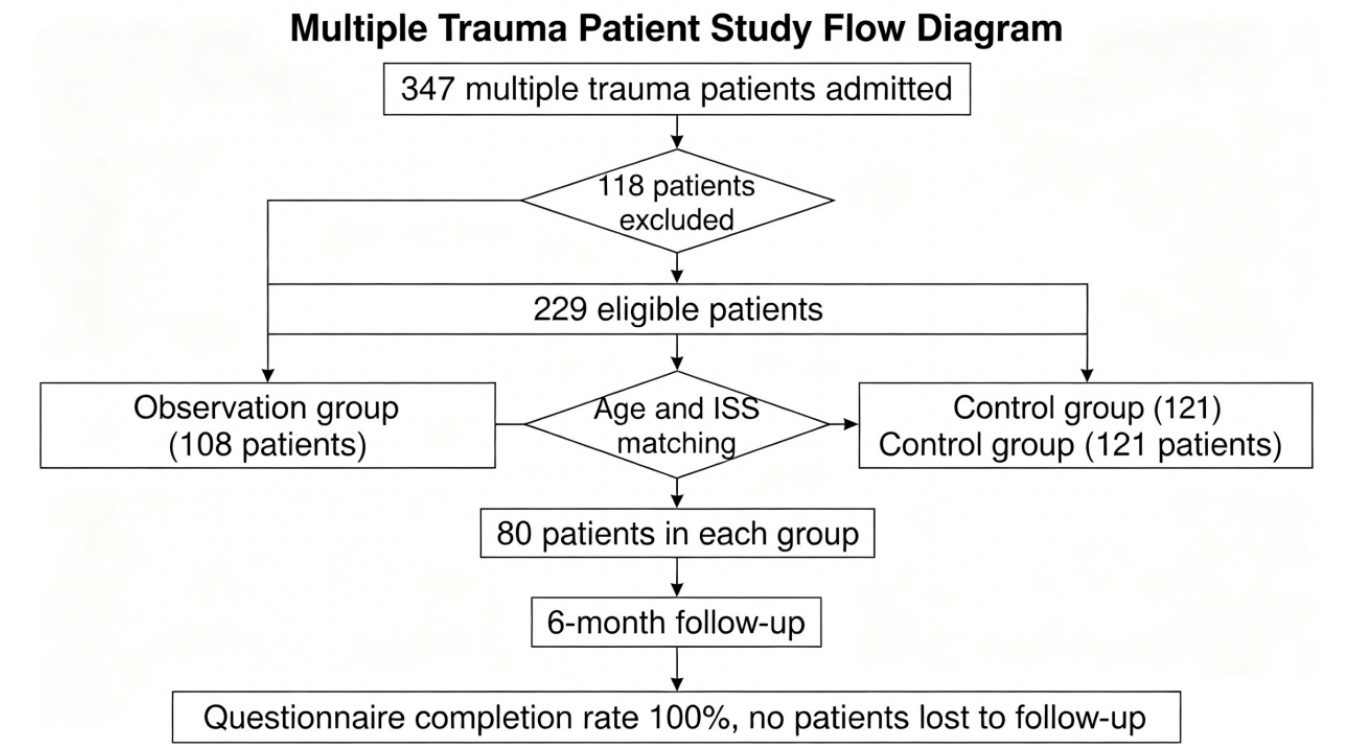

Supplement: Supplementary file 1 [file Image_1.tif]
